# Metropolitan Hospitals and Vivisection

**Published:** 1906-03-03

**Authors:** 


### HOSPITAL ADMINISTRATION.

### CONSTRUCTION AND ECONOMICS.

### METROPOLITAN HOSPITALS AND VIVISECTION.

Mr. Stephen Coleridge has sent us what he denominates on the title-page as "A Guide for the Charitable in the Disposition of their Gifts and Bequests." He appears to be obsessed with wrong-headedness in his zeal for a perfectly legitimate object in itself. This, if promoted by wholly legitimate methods, based upon temperate and restrained discussion, resting on ascertained and proved facts, would have the support of the majority of reasonable people in this country at any rate.

This "Guide for the Charitable in the Disposition of their Gifts and Bequests," of which the present is the 8th edition and the 63rd thousand attempts to hold up to reprobation and discredit several of the hospitals which have medical schools attached to them, on the ground that the financial transactions of many of the Metropolitan Hospitals, as revealed by Sir Edward Fry's commission, are grossly irregular. There is not a word in the preface or letterpress to show that the Council of King Edward's Hospital Fund and the Metropolitan Sunday Fund, both of which are attacked have in fact taken effective measures to secure, that not one penny of the money which the metropolitan hospitals may receive, in future, in grants from one or both of these Funds, will be or can be devoted, to any other object, than the relief of the sick poor. If Mr. Stephen Coleridge had stated this fact, and it is a fact which is incontrovertible, there would have been little if any justification or necessity for him to issue an eighth edition of his so-called "Guide," because the main object of that "Guide" would appear from Mr. Coleridge's remarks to be to secure this result, which has actually been obtained through the independent action of the Council of King Edward's Hospital Fund which appointed Sir Edward Fry's Commission.

Of course Mr. Stephen Coleridge puts in a few strong words upon "the malign influence that vivisection and those who practise it have exercised in undermining the high traditions of these great institutions (our hospitals), and in degrading these ancient haunts of open and beautiful charity into arenas of secret, sordid and brutal research." Mr. Stephen Coleridge is never tired of declaring that he has no desire to injure the hospitals, quite the contrary, but it is "Pretty Fanny's" way of rendering such help to describe our hospitals as "arenas of secret, sordid and brutal research." Mr. Coleridge must know very well, that no man of temperate judgment with a knowledge of the facts, would permit himself to make a statement of this kind, because it is a burlesque of the facts, and is wholly opposed to the truth, so far as the great Metropolitan Hospitals are concerned.

We have always maintained that the medical schools, in regard to the cost of the education of the students, should be put on the same footing as all other schools of training and instruction for students in each of the learned professions. It is only right and proper that the fees charged to medical students shall be inclusive and adequate, and that they shall in fact represent the whole cost of the education and training which are essential to enable an undergraduate to take his degree, and procure the diplomas which will qualify him to engage in the practice of medicine and surgery. Sir Edward Fry's commission has resulted in beneficial reforms in this respect. It has enabled King Edward's Hospital Fund and the Metropolitan Hospital Sunday Fund to give their subscribers a guarantee that all the money entrusted to them shall henceforth be devoted entirely to the relief of the sick in the Voluntary Hospitals of the Metropolis. Mr. Stephen Coleridge must be perfectly well aware that this result has now been secured, and he should accept the fact as an honourable man, instead of taking the unwarrantable course he has done in issuing this eighth edition of a "Guide" which cannot fail, from the way in which it is drawn up, to give the impression to all who are not familiar with the facts, that large sums of money are diverted from the gifts of the public to the hospitals for the relief of the sick poor, to the support of the medical schools, to the maintenance and continuance of vivisection, and, to quote his own words "that these venerable and sacred institutions . . . consecrated in the imagination of mankind to deeds of mercy, and dedicated to the glory of God through the perpetual service of His poor," may pass "into the hands of those who do not scruple to pervert their resources from the succour of the afflicted to the support of the laboratories and schools, wherein unoffending dumb creatures are ruthlessly tortured, in the pursuit of a malignant and heartless science."

Such a statement after the publication and issue of Sir Edward Fry's Report and the steps recently taken, in consequence, by the hospitals, through the action of King Edward's Hospital Fund and the Metropolitan Hospital Sunday Fund, is in fact a shameful falsehood which must reflect the greatest discredit upon everybody who is responsible for its circulation amongst the supporters of hospitals and the public generally. It can only impose upon the ignorant, and we hope that the circulation of documents which are as dishonouring and discrediting to those responsible for their issue, as they are unjust to the hospitals and the great Distributing Funds, may be discontinued. The maintenance of truth and of decency and fair dealing in the public discussion of great questions of paramount importance demands the exhibition of self-restraint and judgment on the part of every man who aspires to lead public opinion or to influence and control it. Will Mr. Stephen Coleridge ever learn and appreciate this fact and act upon it?

